# The preparation of three-dimensional flower-like TiO_2_/TiOF_2_ photocatalyst and its efficient degradation of tetracycline hydrochloride

**DOI:** 10.1039/d1ra01772a

**Published:** 2021-04-21

**Authors:** Chentao Hou, Huayang Liu, Yijie Li

**Affiliations:** College of Geology and Environment, Xi'an University of Science and Technology Xi'an 710054 China 807484470@qq.com

## Abstract

A kind of high-efficiency photocatalyst of the three-dimensional flower-like TiO_2_/TiOF_2_ was synthesized by a one-step hydrothermal method. XRD, FE-SEM, EDS, HTEM, BET, XPS, PL, and UV-Vis-DRS were utilized to characterize the photocatalyst. The photocatalyst of TiO_2_/TiOF_2_ shows a narrow band gap of 2.8 eV. The generation of Ti^3+^ and an oxygen vacancy (O_v_) in the photocatalyst are helpful to increase the absorption of visible light, and to inhibit faster charge recombination by capturing photogenerated carriers. Through the degradation of tetracycline hydrochloride (TCH) under simulated sunlight, the photocatalytic activity and stability of the synthesized samples were investigated. The results showed that the removal rate of tetracycline hydrochloride was 59% only in 0.5 h of dark reaction and 85% in 0.5 h of simulated sunlight. The removal efficiency of the photocatalyst for the adsorption and photocatalytic degradation of TCH is higher than that of the single TiO_2_, TiOF_2_, and Degussa P25. The synthesized three-dimensional flower-like TiO_2_/TiOF_2_ has great application potential in the treatment of antibiotic wastewater.

## Abstract 2

The efficient photocatalytic degradation of tetracycline hydrochloride by three-dimensional flower-like TiO_2_/TiOF_2_.

## Graphical abstract

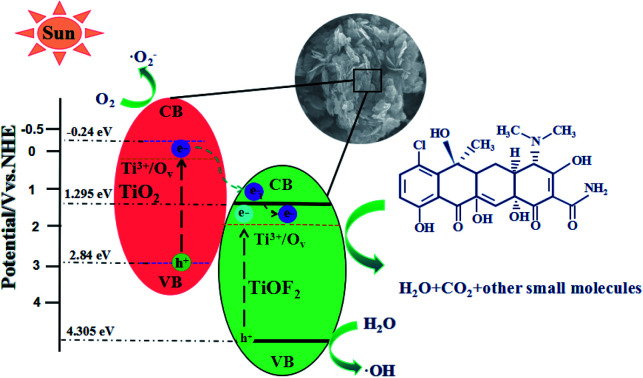

## 1. Introduction

Drugs are a new kind of water pollutant, which is closely monitored by researchers in the aspect of ecology and human health.^1–3^ Antibiotics, as a kind of medicine for the treatment and prevention of bacterial infection, have been widely used in the world, but their risks have been gradually revealed.^4–6^ Over the years, and until now, almost all known symbiotic bacteria and pathogenic bacteria have acquired the ability to produce resistance to one or more antibiotics.^7,8^ Compared with aerosols and soil, water phase transport is considered to be the main way for antibiotics and antibiotic resistance to spread to remote areas and society. Therefore, the treatment of antibiotic wastewater is urgent.

Tetracycline hydrochloride (TCH) is a typical tetracycline broad-spectrum antibiotic, which is widely used as a growth promoter in the treatment of human diseases and animal feeding.^9^ However, because the naphthol ring in the tetracycline hydrochloride structure cannot be completely metabolized by humans and animals, a large amount of absorbed TCH is excreted into various water bodies through feces and urine.^10^ At present, they can be detected in soil, sediment, groundwater, surface water and even drinking water, thus posing a significant risk to the ecosystem and human health. Therefore, the removal of TCH residues from the water environment is very important for the ecological environment and human health.

In recent years, several methods for treating wastewater-containing TCH have been studied, such as microbial degradation,^11,12^ adsorption,^2,5,9^ electrochemical method,^13^ and photocatalytic degradation.^6,7^ Although these methods have a certain removal effect on TCH in wastewater, most of them have some problems. Due to the antibacterial property of TCH, microorganisms do not easily survive in the process of biodegradation, and traditional microbial degradation methods (such as the activated sludge method) cannot effectively remove TCH residues in wastewater.^14^ Although adsorption is one of the most effective methods to remove antibiotics in water, it can only transfer antibiotics from the liquid phase to solid phase, and cannot completely degrade antibiotics.^15,16^ Due to its high energy consumption and poor circulation ability, electrochemical degradation greatly limits its application.^17^ Among these methods, photocatalytic water purification technology is considered to be the most promising water treatment technology due to its cost-effectiveness, efficiency and environment friendliness.^18–20^

TiO_2_, as the most commonly used semiconductor photocatalyst, is nontoxic and chemically stable.^5,7^ However, it has a wide energy band gap (3.0–3.2 eV, which means it only reacts to UV) and a high carrier recombination rate.^21,22^ So far, much effort has been made to make up for these shortcomings, such as metal doping,^23^ non-metal doping,^24,25^ and building a heterogeneous semiconductor composite.^26–28^ However, there are some problems in metal doping, such as thermal instability, toxicity, and high cost.^29,30^ Nonmetallic doping and heterogeneous semiconductor composite materials can effectively improve the separation efficiency of the catalyst carrier, which is recognized as an effective method to improve the performance of the TiO_2_ catalyst.^31^ At present, the construction of a heterojunction mostly needs to prepare the two semiconductors separately, and then form the heterostructure in various ways. This preparation method not only consumes time, but also produces high-energy consumption, which is not conducive to practical production and environmental protection. Therefore, it is very important to find a simple method to prepare a TiO_2_-based heterojunction.

The existence of fluorine could induce Ti^3+^ and Ti–F–Ti bonds to improve the photocatalytic property.^29,32^ The appearance of Ti^3+^ is usually accompanied by oxygen vacancies, which form a continuous vacancy band of electronic states below the conduction band of TiO_2_.^33^ This effectively reduces the recombination of carriers, and makes TiO_2_ respond to visible light. Interestingly, when TiO_2_ is in a high concentration F-ion environment, TiOF_2_ can be formed.^34^

More and more researchers are paying attention to TiOF_2_ photocatalytic materials. According to previous reports, TiOF_2_ has two crystal forms, corresponding to no. 08-0060 and no. 01-0190 in the JCPDS database. The TiOF_2_ corresponding to JCPDS no. 08-0060 shows regular cubic morphology, while the TiOF_2_ corresponding to JCPDS no. 01-0490 shows irregular morphology of nanoparticles. In previous studies, large cubic TiOF_2_ has been widely studied. However, few people have explored the smaller size TiOF_2_ nanoparticles. For example, Dong *et al.*^35^ synthesized the Ag_3_PO_4_/TiOF_2_ composite with cubic TiOF_2_, which improved the photocatalytic activity of Ag_3_PO_4_. Chen *et al.*^36^ used cubic TiOF_2_ as a precursor, through the solid calcination strategy and one-step topological transformation, constructing TiO_2_ hollow nanocubes, and reduced graphene oxide hybrids with a 3D/2D heterojunction to degrade rhodamine B efficiently. Liu *et al.*^37^ found that different hydrothermal synthesis temperatures have a significant effect on the physical and chemical properties, and the photocatalytic activity of TiO_2_/TiOF_2_ nanocomposites. In our previous studies, we used cubic TiOF_2_ as a precursor and synthesized it with Cu_2_O to synthesize the Cu_2_O@TiOF_2_/TiO_2_ composite, which can degrade TCH under simulated sunlight.^38^ However, in our previous research, we did not pay attention to the influence of the different amounts of HF on the formation of the TiO_2_/TiOF_2_ composite. In the preparation process, we need to prepare the cubic TiOF_2_ and Cu_2_O photocatalysts, and then composite them, which greatly increases the manufacturing cost of the photocatalyst. Wang *et al.*^39–41^ explored how different reaction times, different temperatures, and different hydrofluoric acid contents have different effects on the photocatalytic hydrogen production of the {001} surface TiO_2_. They found that TiO_2_ and TiOF_2_ seem to have a synergistic effect. However, they did not continue to explore what reaction conditions could enhance this synergy. Therefore, it is necessary to further reveal the effect of different amounts of HF on TiO_2_, and by controlling the dosage of HF, it is expected that a TiO_2_/TiOF_2_ photocatalyst with Ti^3+^/O_v_ can be synthesized in a one-step hydrothermal method.

In this paper, we discussed the influence of different hydrofluoric acid dosages on the formation of TiO_2_/TiOF_2_, and synthesized a kind of three-dimensional flower-like TiO_2_/TiOF_2_ nanocomposite by one-step hydrothermal method at low temperature for the first time. The flower-like TiO_2_/TiOF_2_ composite has partial oxygen vacancies and a narrow band gap of 2.8 eV, which can respond well to the sun. The photocatalytic activity of the photocatalyst was tested by degradation of tetracycline hydrochloride. The results show that the photocatalyst has a strong adsorption capacity for tetracycline hydrochloride, and shows good photocatalytic activity. Besides, the photocatalyst has good stability, and is expected to be used in the treatment of antibiotic wastewater.

## 2. Experimental section

### 2.1. Reagents and chemicals

Tetrabutyl titanate (TBOT, A. R.), anhydrous ethanol (C_2_H_5_OH, A. R.), *p*-benzoquinone (PBQ), methanol (MT), 1,4-terephthalic acid (PTA), and hydrofluoric acid (HF, A. R.) were purchased from Fuchen Chemical Reagent Factory, Tianjin, China. Tetracycline hydrochloride (TCH) was purchased from Aladdin Industrial Corporation (Shanghai, China). All reagents were used without purification. Ultra-pure water was used as experimental water.

### 2.2. Preparation of the photocatalysts

The TiO_2_/TiOF_2_ heterostructure semiconductor composite was synthesized by a one-step hydrothermal method. 40 mL C_2_H_5_OH was added to 100 mL polytetrafluoroethylene liner, and then the required amount of HF (4 mL, 6 mL, 8 mL, 10 mL) was slowly add under low-speed magnetic stirring at 25 °C. The mixture was mixed at 25 °C for 10 min, and recorded as solution A. 20 mL TBOT was added to solution A under stirring at the rate of 2 drops per second to form a white suspension, and the mixture was stirred at 25 °C for 2 h. Then, the inner liner of polytetrafluoroethylene was placed in a high-pressure reactor and reacted at 150 °C for 15 h. After the system was naturally cooled to room temperature, the solid products were collected by centrifugation, and washed with ethanol and ultra-pure water three times. The samples were dried at 60 °C and named *x*-TF (*x* = 4, 6, 8, 10). For comparative study, we added 4 mL ultra-pure water instead of hydrofluoric acid to prepare pure TiO_2_, and prepared pure TiOF_2_ according to previous reports.^42^

### 2.3. Characterization

The crystal structure of the sample was analyzed by X-ray diffraction (XRD, GD-XD-2, China) equipped with Cu-Kα X-ray source (*λ* = 0.15418 nm). SEM and EDS (JSM7500F, Japan) were used to record the surface morphology and element distribution of the samples. Transmission electron microscopy (TEM) images were achieved with an FEI Tecnai G2 F20, USA. The absorption characteristics of the samples were determined by UV-visible diffuse absorption spectroscopy (UV-Vis DRS, Shimadzu UV-2600, Japan). The photoluminescence spectrum of the photocatalyst was measured by a fluorescence spectrometer (Shimadzu-RF-6000, Japan) with an excitation wavelength of 300 nm. The chemical valence states of the samples were analyzed by X-ray photoelectron spectroscopy (XPS). The specific surface area and porosity of the samples were measured by the N_2_ adsorption–desorption specific surface analyzer (Bet, Micrometrics ASAP 2020, USA).

### 2.4. Photocatalytic tests

The photocatalytic activity of the catalyst was tested by the degradation of TCH. 100 mL of TCH solution (10 mg L^−1^) in a measuring cylinder was put into a 150 mL photocatalytic reaction tube, and then 30 mg of photocatalyst was weighed and added to the TCH solution. The photocatalytic reaction tube was placed into the photocatalytic reactor, and a 500 W xenon lamp was used to simulate the degradation of TCH by sunlight. Before the xenon lamp was turned on, the solution was magnetically stirred in the dark for 30 min to ensure the adsorption–desorption balance. Then, the xenon lamp was turned on to start the photodegradation test. An aliquot of 7 mL suspension was taken out every 5 min, and centrifugated at high speed to remove the catalyst. Then, the remaining TCH concentration was analyzed with a UV-Vis spectrophotometer at the maximum absorption wavelength of 357 nm. To explore the degradation ability of 8-TF to TCH under visible light, a 420 nm filter was added to the photocatalytic reactor, and the above photocatalytic experiment was repeated. Different scavengers (PBQ, PTA, MT) were used to trap the active components (˙O_2_^−^, ˙OH, h^+^) in the photocatalytic process. This test is similar to the photocatalytic degradation test, except that a certain amount of scavenger was added to the TCH solution, and then photocatalyst was added. Finally, the photocatalyst was separated from the TCH solution, and then the next operation was started to study the durability of the catalyst.

## 3. Results and discussion

### 3.1. Crystal structure analysis

**Fig. 1:**
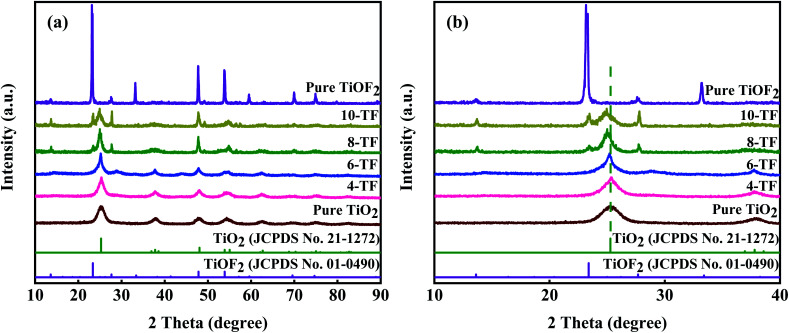
(a) XRD patterns of the prepared samples, and (b) the enlarged XRD patterns of the prepared samples.

Fig. 1a clearly shows the crystal structure of the prepared sample. The diffraction peaks of the samples are very sharp, which shows that the crystal of the material is good. For pure TiOF_2_, the diffraction peaks at 2*θ* = 13.59°, 23.44°, 27.42°, 33.86°, 47.8°, 53.92°, 59.6°, 69.6°, and 74.7° match with those of TiOF_2_ (JCPDS no. 01-0490), indicating that we successfully prepared hexagonal TiOF_2_.^42^ For pure TiO_2_, the diffraction peaks at 2*θ* = 25°, 37.76°, 47.8°, 54.86°, 56.6°, 70.3°, 75°, and 82.22° are attributed to the {101}, {004}, {200}, {105}, {211}, {220}, {215}, {224} planes of anatase TiO_2_ (JCPDS no. 21-1272), respectively.^10^ However, the XRD patterns of 4-TF and 6-TF do not show the diffraction peak of TiOF_2_, which may be due to the low concentration of the F atom. The XRD patterns of 8-TF and 10-TF show the combination characteristics of anatase TiO_2_ and TiOF_2_, which show that we have successfully prepared the TiO_2_/TiOF_2_ composite and the peak intensity of TiOF_2_ increases with the increase of HF. Moreover, no additional crystal phase was observed in all of the spectra, indicating that no impurity was produced in the preparation process.

Fig. 1b shows the amplification comparison of the X-ray diffraction patterns of the prepared samples. It is worth noting that the diffraction peaks on the surface of the TiO_2_ anatase {101} crystals distributed in 8-TF and 10-TF deviate slightly to the low diffraction angle. According to Bragg's law, the distance between D and the diffraction angle is inversely proportional to 2*θ*. Therefore, the negative displacement of the TiO_2_ anatase {101} peaks in 8-TF and 10-TF can be called the broadening of the D-gap, which reveals the interaction between TiO_2_ and TiOF_2_.^43^ The average grain sizes of 4-TF, 6-TF, 8-TF and 10-TF are 18, 15, 13, and 9 nm, respectively, by Scherrer formula

1 *D* = *Kλ*/*βχ* cos *θ*,

where *K* is a constant (the shape factor, about 0.89, *λ* is the X-ray wavelength, *β* is the FWHM of the diffraction line, and *θ* is the diffraction angle).^42^ Moreover, the average grain sizes of pure TiOF_2_ and pure TiO_2_ were calculated to be 26 nm and 22 nm, respectively.

### 3.2. Morphology analysis of the photocatalyst

**Fig. 2:**
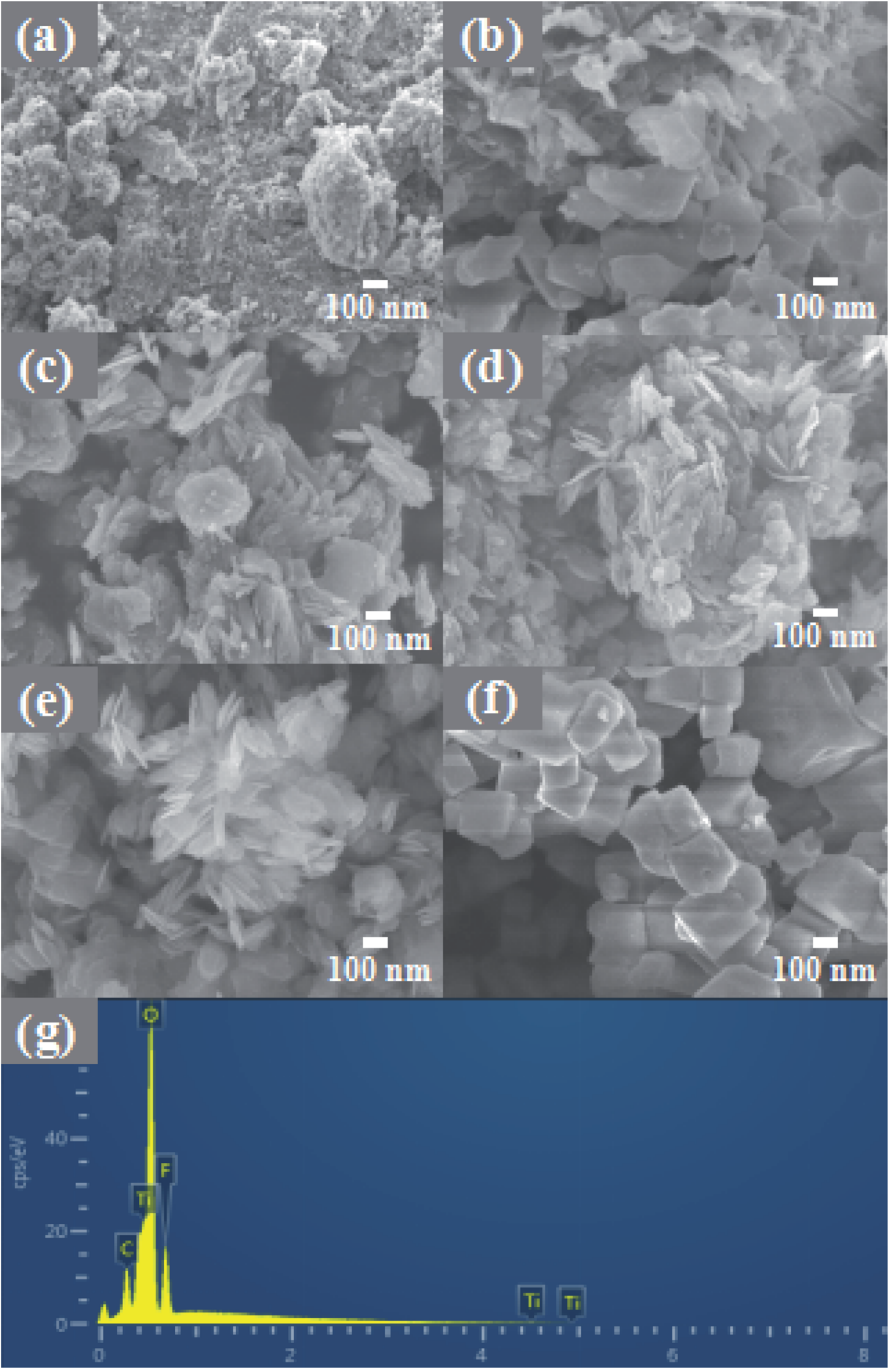
The high-magnification FE-SEM images of (a) TiO_2_, (b) 4-TF, (c) 6-TF, (d) 8-TF, (e) 10-TF and (f). TiOF_2_; EDS images of 8-TF (g).

The morphology and composition of the material were analyzed by SEM and EDS. As shown in Fig. 2a, pure TiO_2_ is composed of nanospheres with a size of 100–500 nm and a relatively rough surface. Fig. 2b shows an image of the product formed by adding 4 mL HF. Under the etching of a small amount of HF, irregular TiO_2_ nanoflakes of 300–500 nm were formed. It can be seen that these nanoflakes are thick and relatively smooth. With the increase of the hydrofluoric acid content, the etching effect is more obvious.

As shown in Fig. 2c, with the further increase of the HF content, most of the thicker and some thinner nanoflakes are generated, which are closely stacked, making the surface somewhat rough. As shown in Fig. 2d, when the HF content reaches 8 mL, the nanoflakes formed are thinner than before, and the nanoflakes are more aggregated, forming a flower-like structure, and obvious mesopores can be seen. This unique morphology with a large specific surface area can provide a large number of reactive sites for pollutants. The measured higher BET specific surface area (67.74 m^2^ g^−1^) is consistent with SEM observation. Interestingly, the XRD pattern of 8-TF has an obvious diffraction peak of TiOF_2_, but there is no image of TiOF_2_ in the SEM pattern. We speculate that the content of 8 mL HF is not enough to react to form a large amount of TiOF_2_, and part of the generated TiOF_2_ has close interface contact with TiO_2_, so it does not appear. Fig. 2e shows the image of the product generated by adding 12 mL HF to the reaction. At this time, compared with the previous nanoflakes, the size is smaller and thinner, the nanoflakes are more dispersed, and the surface tends to be smooth. Fig. 2f shows the image of pure TiOF_2_, showing a small irregular hexagonal structure. Fig. 2g is the EDS spectrum of the 8-TF. The weight percentages of Ti, O, and F in the 8-TF are 66.24%, 27.27%, and 6.49%, respectively.

**Fig. 3:**
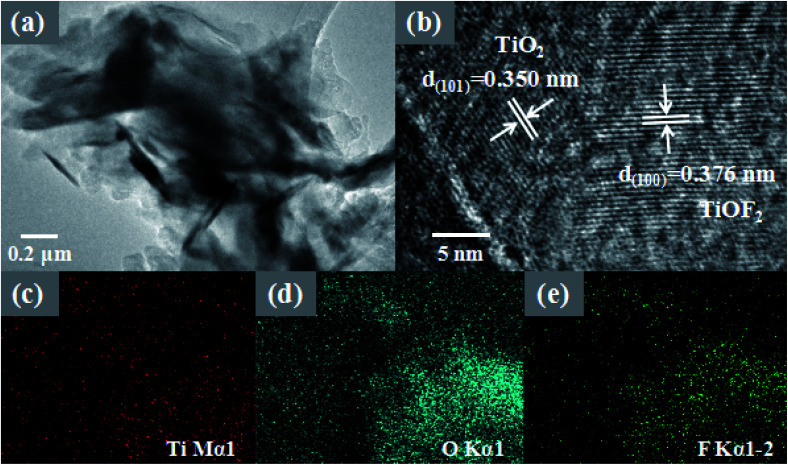
TEM and HRTEM images of 8-TF (a and b); elemental mapping of 8-TF (c–e).

More morphological details on 8-TF were obtained using transmission electron microscopy (TEM) images. It can be seen from Fig. 3a that 8-TF is composed of irregularly shaped nanoflakes, which is consistent with the results observed by SEM. The HR-TEM image of 8-TF showed the intimate contact between TiO_2_ and TiOF_2_ (Fig. 3b). The lattice spacing of *ca.* 0.350 nm can be assigned to the (101) plane of anatase TiO_2_, and the lattice spacing of *ca.* 0.376 nm can be assigned to the (100) plane of TiOF_2_.^40,44^ To further describe the element distribution of 8-TF, elemental mapping analysis (Fig. 3c–e) of 8-TF was performed. The distribution of Ti, O, and F is uniform. These results confirm that we have successfully synthesized a three-dimensional flower-like TiO_2_/TiOF_2_ nanocomposite.

### 3.3. BET specific surface area and pore structure

**Fig. 4:**
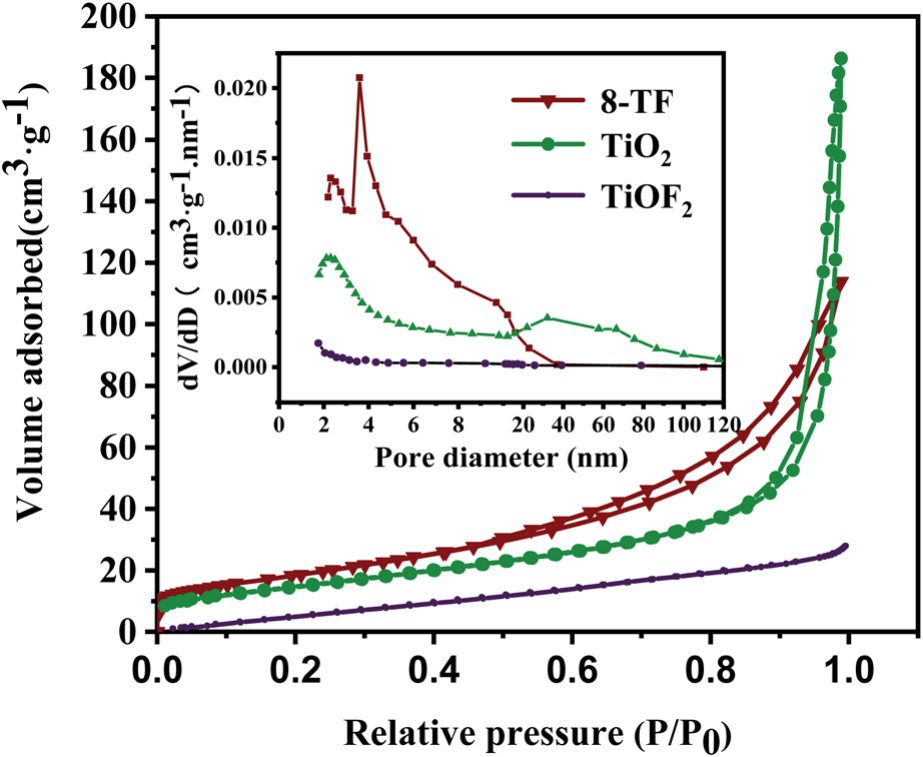
Nitrogen adsorption and desorption isotherms of 8-TF, pure TiOF_2_, and pure TiO_2_. The inset is the corresponding pore size distribution.

The specific surface area and pore size distribution of 8-TF, pure TiO_2_, and pure TiOF_2_ were analyzed by nitrogen adsorption–desorption technology. As shown in Fig. 4, according to the classification of IUPAC, all samples show a typical IV type adsorption desorption isotherm with H3 type hysteresis ring, which represents that the sample has a mesoporous formation and matches the image presented by SEM.^38^

According to the adsorption–desorption isotherm, the specific surface areas of pure TiOF_2_, TiO_2_, and 8-TF are 4.8, 53.6, 67.74 m^2^ g^−1^, respectively. The specific surface area of the composite photocatalyst is significantly higher than that of TiOF_2_ and TiO_2_. The larger specific surface area is beneficial to provide more active sites during photocatalysis, and promotes the degradation of TCH on the surface of the photocatalyst. Table 1 shows the specific surface area, pore-volume, and average pore size for the tested samples. The average pore size of pure TiOF_2_, TiO_2_, and 8-TF is 15.95, 18.69, and 8.56 nm, respectively.

**Table 1:** Specific surface area, volume, and pore size for the samples

Samples	Surface area (m^2^ g^−1^)	Pore volume (cm^3^ g^−1^)	Average pore size (nm)
TiO_2_	53.6	0.28713	18.69
TiOF_2_	4.8	0.023123	15.95
8-TF	67.7	0.18628	8.56

According to the corresponding BJH pore size distribution diagram (Fig. 4), the pore size distribution curve of the 8-TF composite is sharper than that of TiOF_2_ and TiO_2_ alone, indicating that the pore size distribution in the sample is more uniform. Considering the size of the TCH molecule (1.41 nm in length, 0.46 nm in width, and 0.82 nm in height), we think that the 8-TF composite has good adsorption performance. This is consistent with the strong removal rate of the dark reaction stage in the subsequent photocatalytic degradation experiment. Moreover, these uniform and small pore size mesopores are conducive to the absorption of light and multiple reflections inside the material, and provide an effective transmission path for photogenerated carriers.^45^

### 3.4. XPS analysis

**Fig. 5:**
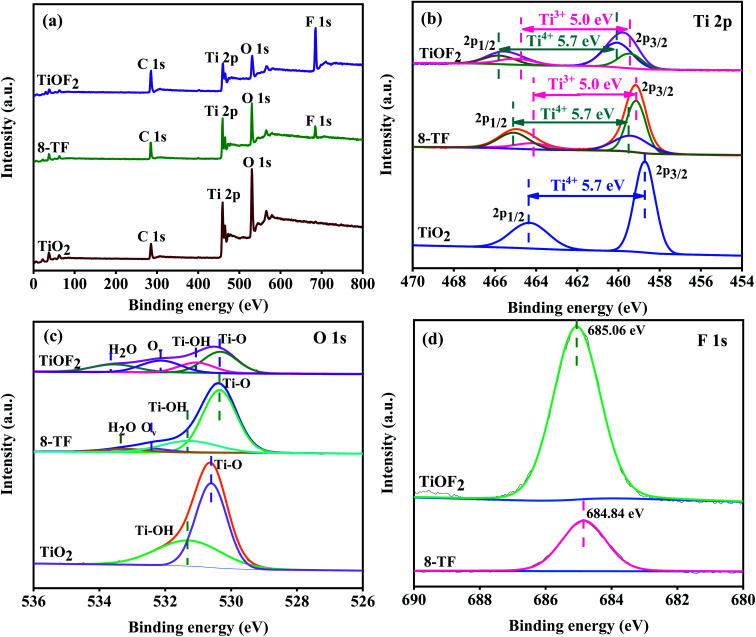
(a) Survey XPS spectra of the samples; (b–d) high-resolution XPS data of Ti 2p, O 1s, and F 1s for the samples, respectively.

The surface compositions and chemical states of pure TiO_2_, TiOF_2_, and 8-TF are displayed in Fig. 5. The survey spectra reveal signals of Ti, O, F, and C on 8-TF that match their respective signals from the individual spectra of TiOF_2_ and TiO_2_ (Fig. 5a), which follows the above EDS results. The existence of the peaks of element C at 284.8 eV can be ascribed to surface adventitious reference carbon, which is unavoidable during the XPS measurement.^44^ From the high-resolution XPS spectrum of Ti 2p in the samples (Fig. 5b), the shape of all of the spectra, being similar to those reported by others, featured a strong peak at about 458 eV and a weak peak at about 464 eV, which were marked as Ti 2p_3/2_ and Ti 2p_1/2_, respectively.^42^ Through Gauss fitting, only 458.68 and 464.38 eV can be observed for pure TiO_2_, corresponding to Ti^4+^ 2p_3/2_ and Ti^4+^ 2p_1/2_, respectively. The distance between the two signal peaks is 5.7 eV, which is strong evidence for the existence of Ti^4+^, indicating that a part of Ti in TiO_2_/TiOF_2_ exists in the form of Ti^4+^.^46^ It is worth noting that the two main peaks of Ti 2p_3/2_ and Ti 2p_1/2_ could be fitted to four peaks after the Gauss fitting of 8-TF and TiOF_2_. For 8-TF, the peaks of 459.2 and 464.2 eV are attributed to Ti^3+^ 2p_3/2_ and Ti^3+^ 2p_1/2_, the peaks of 459.3 and 464.99 eV are attributed to Ti^4+^ 2p_3/2_ and Ti^4+^ 2p_1/2_, respectively, which indicate that Ti^3+^ has formed in the reduction process. The distance between the two signal peaks is 5.0 eV, which is strong evidence for the existence of Ti^3+^, indicating that a part of Ti in TiO_2_/TiOF_2_ exists in the form of Ti^3+^. For TiOF_2_, the peaks of 460.2 and 465.2 eV are attributed to Ti^3+^ 2p_3/2_ and Ti^3+^ 2p_1/2_, the peaks of 460.08 and 465.78 eV are attributed to Ti^4+^ 2p_3/2_ and Ti^4+^ 2p_1/2_, respectively. The binding energies of Ti 2p_3/2_ and Ti 2p_1/2_ of 8-TF and TiOF_2_ move to the high energy region, respectively, compared with the binding energies of TiO_2_, which may be attributed to the fact that the strength of the Ti–F bond is greater than that of the Ti–O bond.^47^ The O 1s spectra of the samples are shown in Fig. 5c. After Gauss fitting of all samples, it can be seen that the O 1s spectrum of TiO_2_ shows two peaks located at 530.6 and 531.28 eV, which are attributed to the Ti–O bond, and the oxygen of the surface hydroxyl.^47^ Interestingly, for 8-TF and TiOF_2_, the asymmetric O 1s spectra were fitted to four different types of O. The photoelectric peaks at 530.35, 531.28, 532.39, and 533.55 eV corresponds to lattice oxygen (Ti–O), hydroxyl oxygen (Ti–OH), oxygen vacancy (O_v_) and oxygen in adsorbed water (H_2_O), respectively, which can be considered as convincing evidence for the existence of Ti^3+^.^48^ Compared with TiO_2_, the binding energy of TiOF_2_ and 8-TF O 1s moves to the low energy region, which may be attributed to the O_v_ in TiOF_2_ and 8-TF. It has been proved in the photo-induced superhydrophilic TiO_2_ that H_2_O molecules are more easily adsorbed onto the O_v_, which explains why the catalyst surfaces are capable of forming more adsorbed H_2_O.^24,32^ It is well accepted that the presence of Ti^3+^/O_v_ can suppress recombination of photo-generated electron–hole pairs, improve the formation rate of photo-induced hydroxyl radicals, and enhance the visible light response. This is consistent with the solid UV spectrum, PL spectrum, and capture experiment. Fig. 5d shows the F 1s XPS spectra. The binding energy of the F 1s electrons shifted from 684.84 eV for 8-TF to 685.06 eV for TiOF_2_ particles. The slight increase in the F 1s binding energy indicated shifting of the surface Ti–F species on the 8-TF nanoparticles to the Ti–F bonds in the bulk TiOF_2_ particles.^41^

### 3.5. Light absorption performance

**Fig. 6:**
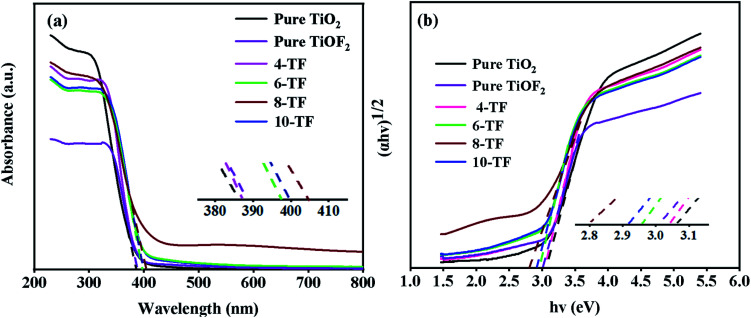
(a) UV-Vis absorbance spectra of the samples, and (b) the corresponding plots of (*αhv*)^1/2^*versus hv*.

The diffuse UV-Vis absorption spectra of pure TiO_2_, pure TiOF_2_, and *x*-TF composites are shown in Fig. 6a. It can be seen that pure TiO_2_ and TiOF_2_ show obvious adsorption in the UV region, but show limited absorption in the visible region. Compared with pure TiO_2_ and pure TiOF_2_, the absorption of the *x*-TF (*x* = 4, 6, 8, 10) composite in the visible light region is significantly enhanced, the absorption threshold is significantly red-shifted, and the absorption edge in the visible light region is significantly increased, which may be due to its special flower cluster structure, close interface contact between TiO_2_ and TiOF_2_, and partial oxygen vacancy in the photocatalyst.

Interestingly, when the dosage of HF is increased from 4 mL to 8 mL, the absorption of visible light by the composite is continuously enhanced. However, when the dosage of HF is increased to 10 mL, the absorption of visible light by the composite is weakened, indicating that the content of TiOF_2_ is too much, which may produce a light-shielding effect. Because 8-TF has strong absorption in the UV region and can absorb part of the visible light, the composite can make greater use of sunlight, which can improve the overall quantum efficiency of pollutant oxidation.^49^ This is consistent with the subsequent degradation test.

The band gap energy (*E*_g_) of pure TiO_2_, TiOF_2_, and *x*-TF was calculated according to the Tauc curve. The results are shown in Fig. 6b. The band gap energies of TiO_2_ and TiOF_2_ are 3.08 and 3.01 eV, respectively, which are close to those reported in the literature.^42,49^ Because of the heterojunction between TiO_2_ and TiOF_2_, the band gap of the *x*-TF composite is narrowed to 3.04 (4-TF), 2.97 (6-TF), 2.8 (8-TF), and 2.92 eV (10-TF), respectively.

### 3.6. Photocatalytic activity

**Fig. 7:**
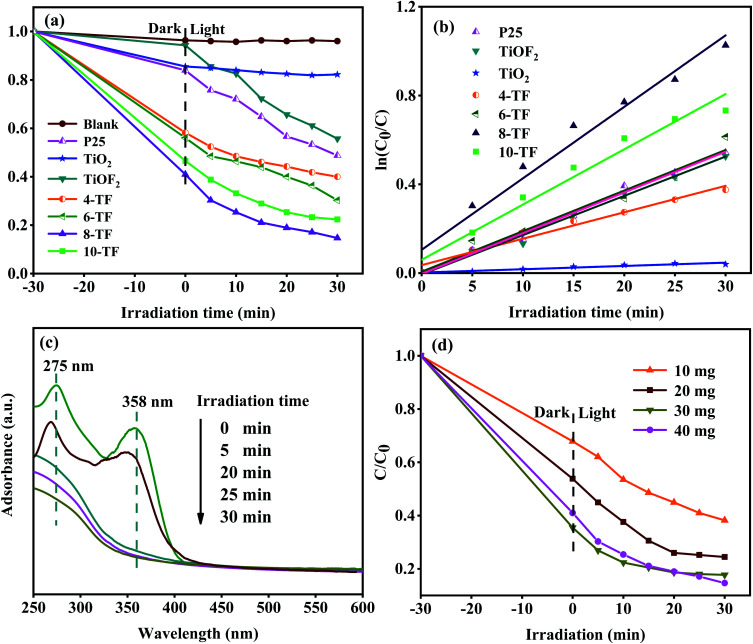
(a) Photodegradation of TCH using different catalysts under simulated solar light; (b) kinetic linear simulation curves of the TCH photodegradation; (c) absorption spectra of the TCH solution collected during the photodegradation of 8-TF; (d) the effect of different amounts of catalysts (10, 20, 30, 40 mg) on the simulated solar light photocatalytic properties of 8-TF.

To study the photocatalytic performance of our samples, tetracycline hydrochloride (TCH) was used as a typical antibiotic pollutant in water to simulate the solar photocatalytic degradation test. The results are shown in Fig. 7a. The photocatalysis system reacts in the dark for 30 min to reach the adsorption–desorption equilibrium before the sunlight exposure. Because TCH is also sensitive to light, for comparison, we carried out a blank experiment without any photocatalyst. TCH has little self-degradation under sunlight. Moreover, the removal rate of pure TiO_2_ for TCH is only 14.5%, which may be attributed to its low specific surface area. This makes it unable to provide more active sites for TCH. This explanation also applies to the pure TiOF_2_ nanoparticles with a low adsorption degradation rate (8%). Due to the low utilization rate of sunlight and high surface charge recombination rate of TiO_2_, the total removal rate of TCH after 0.5 h of sunlight irradiation is only 17.8%. The photodegradation efficiency of TCH (about 44%) by pure TiOF_2_ is also not satisfactory.

We also investigated the photocatalytic activity of commercial titanium dioxide (Degusse P25). The adsorption and degradation of p25 to TCH are consistent with that of pure TiO_2_. After 30 min of photocatalytic action, the total degradation rate of TCH is 51%. The results show that the photocatalytic activity of p25 is higher than that of pure TiO_2_ synthesized in this study. Although the two TiO_2_ particles are similar in morphology and structure, the commercial p25 particles are composed of two crystal types: anatase type and rutile type. Compared with single-phase TiO_2_, P25 is more conducive to the use of visible light.

All of the *x*-TF (*x* = 4, 6, 8, and 10) samples showed significantly enhanced adsorption and photocatalysis performance compared with pure TiO_2_ and TiOF_2_. The adsorption and removal rates of TCH were 42%, 44%, 59%, and 53.4%. The total removal rates were 60%, 70%, 85%, and 77% after 30 min of reaction under sunlight. Among them, 8-TF has the highest total removal rate of TCH. This may be attributed to the fact that 8-TF can easily reach the surface of the catalyst due to its unique flower-like morphology, and its large specific surface area provides more active sites for the photocatalyst, which make it have strong adsorption. Due to the higher absorption of UV and visible light, 8-TF has high electron separation efficiency. The smaller particle size makes its active center density larger. Furthermore, the close heterogeneous interface and the presence of Ti^3+^/O_v_ slow down the electron–hole recombination rate, thus showing good photocatalytic activity.

The degradation kinetic model was studied and the photocatalytic properties of the samples were further analyzed. Fig. 7b shows that the linear relationship between the degradation time (*t*) and ln(*C*_0_/*C*) is almost a straight line, which proves that the degradation process of TCH conforms to the pseudo-first-order reaction.^50^ The rate constants *k* of pure TiO_2_, TiOF_2_, P25, and *x*-TF (*x* = 4, 6, 8, 10) were 0.00149, 0.01771, 0.01837, 0.01192, 0.01829, 0.03222, and 0.0249 min^−1^, respectively. The results show that the rate constants of 8-TF are 21, 1.8, and 1.7 times higher than those of TiO_2_, TiOF_2_, and P25, respectively. The results show that the addition of 8 mL HF is conducive to the formation of the TiO_2_/TiOF_2_ composite with high photocatalytic activity. The absorption spectrum of TCH degraded by 8-TF is shown in Fig. 7c. TCH has two main absorption peaks at 275 and 358 nm. The absorption peak at 275 nm may be related to the hydroxyl and acylamino produced in the reduction process.^51^ The absorption peak at 358 nm can be attributed to the aromatic ring B–D. The absorption peak decreased rapidly after sunlight irradiation. After 30 min of irradiation, the absorption peak almost disappeared, which indicated that the ring structure of TCH was destroyed after the light source was added.

To study the effect of catalyst loading on the photocatalytic degradation system, the experiment was carried out by changing the amount of catalyst. Fig. 7d shows the degradation curve of TCH solution with different 8-TF dosages (0.1 g L^−1^, 0.2 g L^−1^, 0.3 g L^−1^, 0.4 g L^−1^). When the amount of catalyst is 0.3 g L^−1^, the degradation effect of TCH is the best. Although the degradation rate of the 0.4 g L^−1^ catalyst is the same as that of the 0.3 g L^−1^ catalyst, the photocatalytic rate of the 0.4 g L^−1^ catalyst is lower than that of the 0.3 g L^−1^ catalyst, which may be due to the aggregation of excessive catalyst. This makes the solution turbid and leads to photon scattering, thus reducing the photocatalytic rate.^52^

**Fig. 8:**
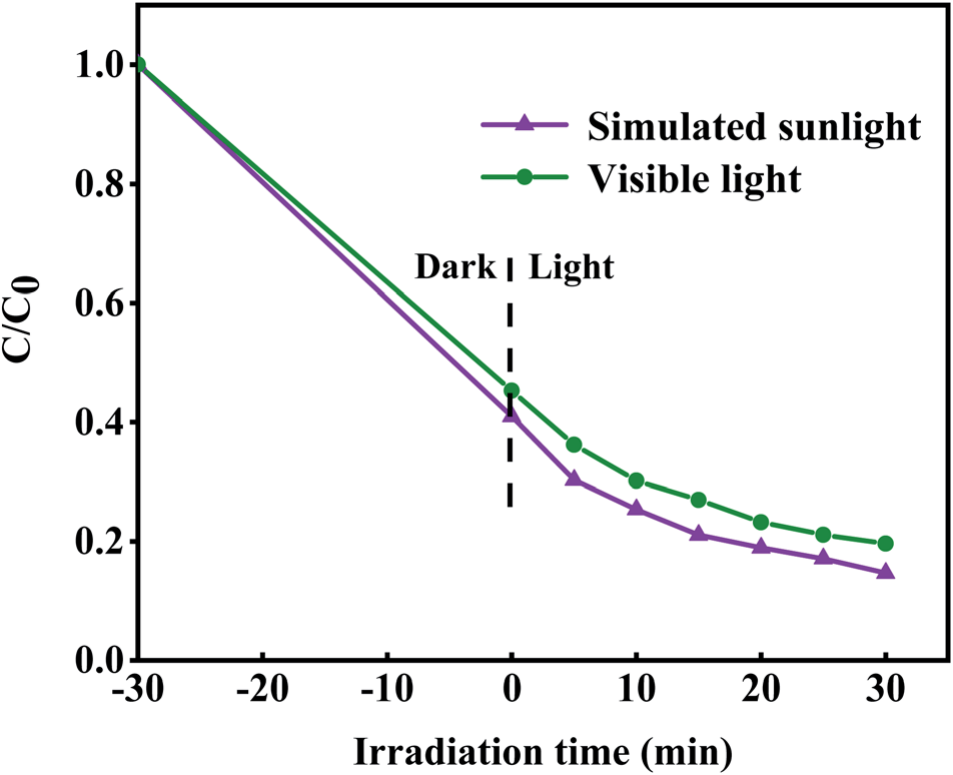
Photodegradation efficiency of TCH by 8-TF under simulated sunlight and visible light (>420 nm) irradiations.

**Fig. 9:**
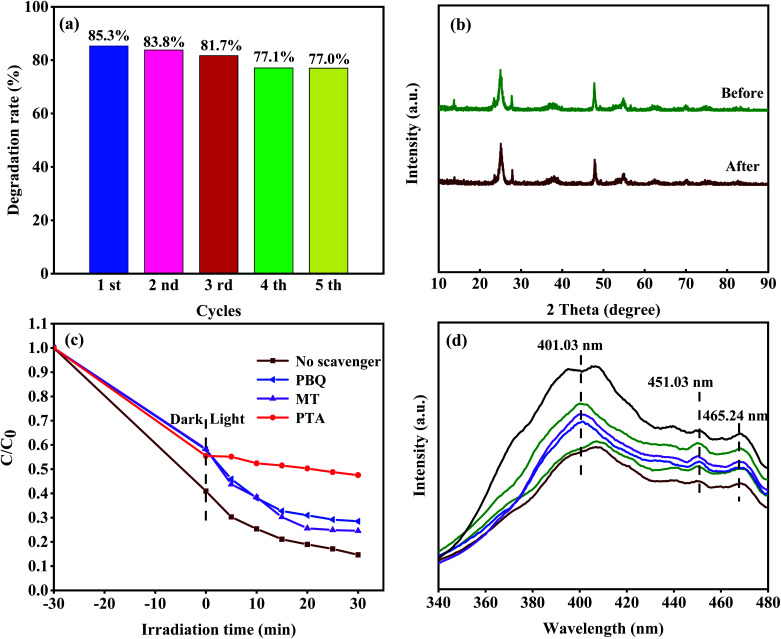
(a) Photostability tests over 8-TF for TCH degradation; (b) XRD patterns of 8-TF before and after five cycling runs; (c) effect of different scavengers on the degradation of TCH over 8-TF; (d) the PL spectra with an excitation wavelength of 300 nm.

Moreover, we used a 420 nm filter to filter the ultraviolet light in simulated sunlight, and tested the photocatalytic activity of 8-TF under visible light (Fig. 8). The results showed that 8-TF still had a good degradation effect on TCH under 30 min visible light irradiation (85%). The stability and reusability of the photocatalyst are important factors affecting its practical application. Therefore, five consecutive cyclic photodegradation tests were carried out on sample 8-TF, and the results are shown in Fig. 9a.

After five cycles, the degradation rate of sample 8-TF in simulated sunlight decreased from 85.3% to 77.0%, indicating that sample 8-TF has a high reuse rate and photocatalytic performance. As shown in Fig. 9b, after five cycles, the peak intensity of the 8-TF photocatalyst decreased, but the position of the diffraction peak was the same as that before the reaction, indicating that the 8-TF heterojunction has high chemical stability.

### 3.7 Photocatalysis mechanism

In the process of photodegradation, the photo-induced electron–hole pair can be separated and transferred to the surface of the photocatalyst, directly participating in the photocatalytic reaction or producing active radicals.^53^ In general, hydroxyl radicals (˙OH), superoxide radicals (˙O_2_^−^), and holes (h^+^) can be found in the photocatalytic degradation process.

For this reason, many scavengers, such as *p*-benzoquinone (PBQ, ˙O_2_^−^), 1,4-terephthalic acid (PTA, ˙OH), methanol (MT, h^+^) and others, were introduced to carry out free radical trapping experiments. It can be seen from Fig. 9c that in the dark reaction stage, the scavenger has a slight influence on the adsorption of the system. We speculate that the photocatalyst has a different degree of adsorption on different scavengers, and the scavengers and TCH have formed a competitive relationship. The combination of different scavenger and catalyst surfaces may also be different, so different catalysts show different adsorption effects in the dark reaction stage. After adding PTA, the degradation efficiency of TCH decreased from 85.3% (no scavenger) to 52.4% (with PTA), indicating that the ˙OH radical is the main active species in the photocatalytic degradation process. When PBQ entered the reaction system, the degradation rate of TCH decreased from 85.3% (no scavenger) to 71.5% (PBQ existed), indicating that ˙O_2_^−^ played a secondary role in the degradation system. When MT was added to the reaction system, the degradation rate of TCH decreased from 85.3% (no scavenger) to 75.4% (MT existed), indicating that h^+^ played a secondary role in the reaction system. Therefore, the contribution order of the active species in the photocatalytic degradation process is ˙OH > ˙O_2_^−^ > h^+^.

The migration of photoexcited e^−^ and h^+^ in the photocatalyst was studied by PL analysis. It is well known that the photoluminescence intensity is closely related to the carrier recombination rate. The PL spectrum (Fig. 9d) of the sample was obtained by the excitation wavelength of 300 nm. The broad emission band at 401.03 nm is considered to be the emission boundary formed by the capture of free excitons by the titanium group near the defect.^44^ The peaks of 451.03 and 465.24 nm can be attributed to oxygen vacancy and electron capture, respectively. It can be seen that the luminous intensity of 8-TF is significantly lower than that of other samples, which indicates that the composite rate of e^−^ and h^+^ is the lowest. This is consistent with the results of the photocatalytic degradation test. This may be due to the close interface contact between TiO_2_ and TiOF_2_, which promotes the transmission of electrons.

The position of the band gap plays an important role in the flow direction of photoexcited electrons and holes. This plays an important role in the production of active species and the performance of photocatalysis. We calculated the location of the conduction band (CB) and valence band (VB) for TiO_2_ and TiOF_2_ using the following empirical formula:

2 *E*_c_ = *χ* − *E*_0_ − 0.5*E*_g_

3 *E*_v_ = *E*_c_ + *E*_g_

here, *E*_c_ is the energy of the conduction band, *E*_v_ is the energy of the valence band. *χ* represents the absolute electronegativity of the semiconductor, and the values of TiO_2_ and TiOF_2_ are 5.8 and 7.3 eV, respectively.^44,49^*E*_0_ is the energy of the free-electron on the hydrogen scale, which is 4.5 eV. *E*_g_ is the band gap energy of semiconductors. According to the DRS analysis above, the band gap energy of TiO_2_ and TiOF_2_ is 3.08 eV and 3.01 eV, respectively. Through calculation, the lead and valence band positions of TiO_2_ and TiOF_2_ are −0.24/2.84 eV and 1.295/4.305 eV, respectively.

**Fig. 10:**
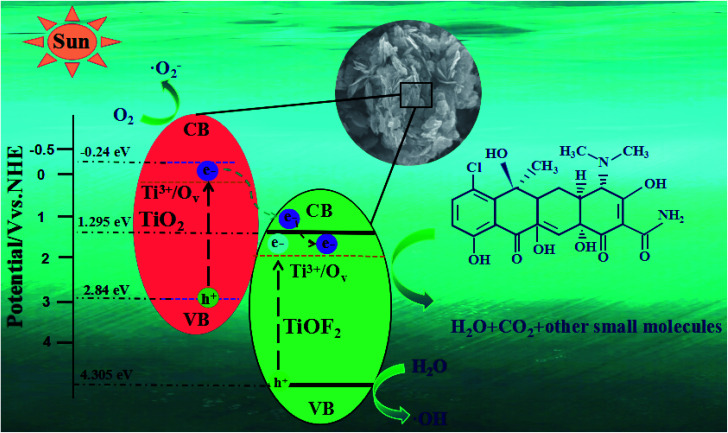
Schematic illustration of the photocatalytic mechanism for TCH removal over the 8-TF photocatalyst.

Based on the experimental results and band gap position, we proposed the possible mechanism of outstanding photocatalytic activity of 8-TF (Fig. 10). First, the specific surface area of the catalyst is greatly increased due to its unique flower cluster morphology. A large specific surface area not only increases the contact area of the reactant adsorption, but also provides more active sites for the photocatalytic reaction.^10^ This unique morphology also makes it have strong light absorption characteristics, which is conducive to improving the quantum efficiency of photons. Due to the existence of Ti^3+^/O_v_, there are local states below the conduction band of TiO_2_ and TiOF_2_, which makes TiO_2_ and TiOF_2_ respond well to simulated sunlight. Moreover, the heterostructure formed by TiO_2_ and TiOF_2_ reduces the recombination efficiency of the electrons and holes, which makes the electrons and holes more likely to interact with O_2_ and H_2_O to generate an active species capable of degrading TCH. Due to the CB potential (−0.24 eV) of TiO_2_ being more negative than that of TiOF_2_ (1.295 eV), a strong driving force will be generated between the close heterogeneous interfaces, which will promote the electron transfer from the CB of TiO_2_ to the CB of TiOF_2_. Due to the existence of some oxygen vacancies in the material, some electrons can be captured. It is obvious that the directional transfer of electrons and the capture of oxygen vacancies effectively reduce the recombination rate of electron–hole pairs, which is the main reason for the excellent photocatalytic activity of the photocatalyst. Because the CB potential (−0.24 eV) of TiO_2_ is more negative than that of O_2_/˙O (−0.046 eV),^54^ the adsorbed O_2_ on the surface of the photocatalyst is more likely to capture the electrons on TiO_2_ CB and form ˙O_2_^−^. ˙O_2_^−^ can highly oxidize TCH or other pollutants. Moreover, because the material has some oxygen vacancies, these oxygen vacancies can better adsorb water, which plays an important role in the formation of hydroxyl radicals. Because the VB potential (4.305 eV) of TiOF_2_ is higher than that of TiO_2_ (2.84 eV), the h^+^ on TiOF_2_ VB is easier to transfer to the VB of TiO_2_, and the VB potential of both is higher than the standard redox potential (1.99 eV) of ˙OH/OH^−^.^55^ This allows the adsorbed water on the surface of the photocatalyst to more easily form ˙OH with strong oxidation and can mineralize TCH well.

## 4. Conclusions

To sum up, a new type of three-dimensional flower-like TiO_2_/TiOF_2_ heterostructure semiconductor composite was successfully prepared by a one-step hydrothermal method. Compared with Degusse P25, TiOF_2_, and pure TiO_2_, the degradation of TCH on the prepared photocatalyst was improved, and the rate constants were 1.7, 1.8, and 21 times higher than that for P25, TiOF_2_ and TiO_2_ respectively. The unique morphology provides a large specific surface area for the adsorption of reactants, and the close heterogeneous interface and Ti^3+^/O_v_ are conducive to charge transfer and separation, reducing the carrier recombination rate. It is worth noting that there are many interference factors in the actual TCH wastewater. Therefore, the interference factors in the actual antibiotic wastewater should be evaluated and tested in the follow-up study.

## Conflicts of interest

There is no conflict of interests existing in the manuscript submission, and it is approved by all of the authors for publication. All the authors listed have approved the manuscript to be enclosed.

## Supplementary Material
